# The Validity of Children’s Fruit and Vegetable Intake Using Plasma Vitamins A, C, and E: The SAYCARE Study

**DOI:** 10.3390/nu11081815

**Published:** 2019-08-06

**Authors:** Tatiana Sadalla Collese, Augusto César Ferreira De Moraes, Tara Rendo-Urteaga, Liania Alves Luzia, Patrícia Helen de Carvalho Rondó, Dirce Maria Lobo Marchioni, Heráclito Barbosa Carvalho

**Affiliations:** 1 YCARE (Youth/Child cArdiovascular Risk and Environmental) Research Group, Department of Preventive Medicine, Faculdade de Medicina, Universidade de Sao Paulo, Sao Paulo 01246-903, Brazil; 2 Department of Epidemiology, School of Public Health, University of Sao Paulo, Sao Paulo 01246-904, Brazil; 3 Facultad de Ciencias de la Salud, Universidad Isabel I, 09001 Burgos, Spain; 4 Department of Nutrition, School of Public Health, University of Sao Paulo, Sao Paulo 1246-904, Brazil

**Keywords:** children, fruits and vegetables, biomarkers, vitamins, validity, food frequency questionnaire, 24-h-Dietary-Recall, Multiple Source Method

## Abstract

Despite that fruits and vegetables are key elements for health promotion, there are limited studies validating their intake in children. We aimed to validate the SAYCARE (South American Youth/Child Cardiovascular and Environmental) Study Food Frequency Questionnaire (FFQ) and the combination of the FFQ frequency of intake with the 24 h-dietary-recall (24 h-DR) (mean of 3 days), for children’s fruit and vegetable intake. The reference methods were plasma dosages of β-carotene, retinol, ascorbic acid, and α-tocopherol, which were collected in the school environment. It is a validity study in a subsample of 45 children aged 6–10 years participating in the SAYCARE Study, from São Paulo (Brazil). The FFQ was answered by the parents/guardians over the previous 3 months; the 24 h-DR was answered three times (two weekdays by nutritionists, one weekend day by parents/guardians). The mean fruit and vegetable intake (combined with frequency of intake) was calculated using the multiple source method (MSM). Multiple linear regression showed pooled correlation coefficients of 0.29 to 0.35 for the reported fruit and vegetable intake estimated by the FFQ and the MSM, respectively. The SAYCARE FFQ is an accurate and useful tool for ranking fruit and vegetable intake in children between 6–10 years from the SAYCARE Study.

## 1. Introduction

Fruits and vegetables are key elements for the promotion of lifelong health [1]. However, there are limited studies validating questionnaires for children’s fruit and vegetable intake [2]. Assessing dietary intake in children is a complex and challenging task [3], because it often relays on the parental report, mainly when children are younger than 10 years of age [4]. As the custom of families eating together is constantly decreasing [5], parental knowledge about their children’s dietary intake is difficult to assess with accuracy [6].

Accuracy is the precision of a measurement or the degree to which a tool measures the aspect it was designed to measure, usually assessed to validate a tool [7]. There are several concepts related to the validity of dietary assessment methods, including the validity of their results against a reference method that has a superior level of validity [8]. In the usual fruit and vegetable intake, for example, the validity of a food frequency questionnaire (FFQ) newly developed might be assessed by comparing it with 24-h dietary recalls (24 h-DR), and with concentration biomarkers. None of these examples is a perfect method, but the validity of the FFQ will be enhanced if there is agreement among the different methods [8].

As all dietary assessment methods have their strengths and limitations, understanding these details may help to apply the dietary assessment methods in the appropriate context [9]. The FFQ and the 24 h-DR are subjective methods widely applied in epidemiological studies, because they are relatively inexpensive and easy to administer, although prone to random and systematic bias [7]. Plasma vitamins can be used as nutritional biomarkers to provide an objective assessment of dietary intake, independently of the errors inherent from the FFQ and the 24 h-DR [10]. Plasma vitamins are a class of concentration biomarkers which are used for comparisons with estimates of fruit and vegetable intake [10]. Concentration biomarkers cannot be translated into absolute levels of fruit and vegetable intake, however, these biomarkers do correlate with fruit and vegetable intake in certain levels [10]. Examples of concentration biomarkers which can be used for ranking fruit and vegetable intake are as follows: Vitamin A (mainly β-carotene, the major source of vegetable pro-vitamin A; and retinol, because most of the β-carotene available is converted into retinol in the blood) [11]; vitamin E [dark-green pigmented leaves and vegetables contain low to moderate levels of α-tocopherol (vitamin E activity)] [12]; and vitamin C (ascorbic acid (vitamin C) contents are found predominantly in citrus fruits, fruit juices, and vegetables) [13,14]. Thus, these plasma vitamins can be applied as a proxy for fruit and vegetable intake, and as a reference method in validity studies of subjective methods ranking fruit and vegetable intake [13].

In spite of that, validity studies ranking children’s fruit and vegetable intake are lacking, particularly in low and middle-income countries [3]. For example, in South American countries, where it is important to improve the classification of children’s fruit and vegetable intake due to the great variety of fruit and vegetable eating options in these regions [15], and due to changing demographical and epidemiological factors [16].

## 2. Objective

To validate the FFQ and the combination of the frequency of intake from the FFQ with the 24 h-DR (mean of three days), both designed for the SAYCARE (South American Youth/Child Cardiovascular and Environmental) Study, for children’s fruit and vegetable intake, using plasma vitamins A, C, and E.

## 3. Methods

### 3.1. Study Design

This is a validity study that is part of the SAYCARE (South American Youth/Child Cardiovascular and Environmental) study. The SAYCARE study is an observational, multicenter, and feasibility study aimed at developing valid and reliable instruments to obtain information about: Social and environmental factors, family environment, food intake and behavior, physical activity and sedentary behaviors, body composition, sleep, oral health and cardiovascular health biomarkers in South American children and adolescents (3–17 years) from seven cities: Buenos Aires (Argentina), Lima (Peru), Medellin (Colombia), Montevideo (Uruguay), Santiago (Chile), and São Paulo and Teresina (Brazil) [17]. Details on the sampling, recruitment, and quality control procedures of the SAYCARE Study were described previously in the SAYCARE Study supplement [17].

### 3.2. Study Population

The research participants were a convenience sample of children drawn from the SAYCARE study (aged 6–10 years), belonging to the survey center of São Paulo (one public and one private school). Children were selected if they donated blood samples for analyses of complete blood count, total cholesterol and plasma vitamins. These children were selected aiming the highest methodological rigor as possible in the fieldwork logistics and, especially, due to the complexity in conducting the blood examinations at the school environment and the transport of biological material (more details are given in Section 3.6. Blood processing and analyzes).The calculation for this sample was based on the experience of validity studies of dietary assessment methods in children [3]. This calculation ideally considered the required parameters for validity studies, such as the two-tailed error of 0.05 (type I), error β of 0.20 (error type II), and correlation coefficient of 0.32 (mean of the correlation coefficients of validation studies of dietary assessment methods in children with plasma vitamins) [3], resulting in a total of 30 children.

This sample was randomly extracted from the general sample of the SAYCARE study, with research participants equally distributed by sex and type of school (public and private). Anticipating losses and refusals of 50%, we invited 60 children to participate (30 from each school).

#### Eligibility Criteria

Inclusion criteria: School children (aged between 6 and 10 years old) enrolled in the selected schools, with signed parental consent form, verbal assent of each child and blood collection performed. Furthermore, children in fasting status (10 h) at the time of collection, and with complete information about sex, age, FFQ, and 3 days of 24 h-DR (with 15 days-interval among each 24 h-DR).

Exclusion criteria: Inability to answer the questionnaires; children with physiologically implausible energy intake for this age group (less than 500 or more than 4000 kcal per day) estimated by the FFQ; children under supplementation with vitamin A, C, and/or E; or children with C-reactive protein concentrations above 10 mg/dL.

### 3.3. Fieldwork

Fieldwork was done according to the schedule and availability of the schools, between August to November 2017.

Several measurement instruments were used in two main groups: Objective measures (anthropometry and plasma vitamins) and subjective measures (questionnaires). Once the schools had all the information about the study and agreed to participate in it, fieldwork occurred on four school visits:

First visit: Meeting with parents/guardians for the presentation of the study and invitation to participate in it, with the delivery of the parental consent form.

Second visit: Once the parental consent form was signed, it was reviewed and signed by the study researchers as well. An original copy of the consent form was delivered to the parents/guardians, together with the questionnaires, in a colorful bag with the identification of the SAYCARE Study. After each child assented to participate in the study, we performed the anthropometric measures and a nutritionist applied the first 24 h-DR. On that day, the children were instructed about the preparation for the blood test, and this information was also posted on the research participants’ agenda. Furthermore, text messages were sent to their parents’ cell phones to remind them of this preparation the day before the blood test.

Third visit: Blood extraction and the second 24 h-DR was applied by a nutritionist (with 15 days-interval from the first 24 h-DR). All the children received a snack after the blood exam.

Fourth visit: The results of the blood tests were evaluated individually by a clinical pathologist and then sent to the parents/guardians (direct mail), along with a letter of appreciation for participation in the study and general guidelines for healthy eating. If any variation was found in these results, an accompanying letter to the health service was attached. Thereafter, the researchers went to the schools to acknowledge the participation of the children (and the collaboration of the teachers and school staff) in the study. Plays were done with the children to explain the importance of fruit and vegetable intake and the main sources of vitamins A, C, and E. For that purpose, we used plastic fruits and vegetables; vitamin A, C, and E in balloon shape; and a hand puppet of a monkey named “Ms. Bananas”. The “Ms. Bananas” was also used to play with kids in the fieldwork, aiming a more interactive and ludic process during their participation in the study, especially during the blood extraction.

### 3.4. Anthropometric Measures

Body composition assessments were performed by trained professionals using the standardized SAYCARE Study methodology [17]. Such assessments were conducted in a reserved room within each school, with children wearing lightweight and barefoot clothing (no socks). The variables analyzed, according to the reference manual of anthropometric standardization of the World Health Organization [18], were:

Weight: Measured with an ultra Slim W801 digital scale, with a maximum capacity of 150 kg, and an accuracy of 0.1 kg (Crivitta Diagnostica Ltd., São Paulo, Brazil).
Height: Measured with a wall stadiometer with a millimeter scale (Cardiomed, Paraná, Brazil).
Waist circumference: Measured with a flexible and inextensible tape (in centimeters) positioned between the narrowest point between the last rib and the upper anterior iliac spine.

Measurements were repeated twice, and a third measurement was performed only in case of error higher than 5% between the first and second measurements. Weight and height were used to calculate body mass index (BMI)—weight (kg) divided by height (meters) squared—and it was classified according to the criteria defined by Cole et al., 2007 [19].

### 3.5. Subjective Dietary Assessment Methods

In order to assist in the estimation of food portions, a food booklet was developed for the SAYCARE Study, with photos of commonly consumed foods (including specific foods from each center participating in the SAYCARE Study) and their standard portion sizes [20]. There are many units of measurement in this food booklet (e.g., spoons, glasses, plates, bowls, bottles, cans), and all have been standardized for the SAYCARE Study. For example, fresh fruit (medium portion 120 g): Orange, apple, banana; fresh fruit juice (with or without sugar and medium portion defined in glasses); raw vegetables (medium portion 50 g): Carrots, beets, tomatoes, cucumbers; Boiled vegetables (medium portion 50 g): Carrot, eggplant, pumpkin, broccoli, cassava, yam; Vegetable soup (average portion of 130 mL). Both FFQ and 24 h-DR (described below) were carried out with the support of this food booklet (delivered with the questionnaires).

#### 3.5.1. Food Frequency Questionnaire (FFQ)

More details on the development and validity of the semi-quantitative FFQ designed for the SAYCARE Study [20] and its validity were described elsewhere [21]. The SAYCARE FFQ refers to usual consumption from the last three months. It was developed considering the national lists of foods commonly consumed by children in each center, and classified according to the food groups from FAO (Food and Agriculture Organization of the United Nations). It consists of a list of 56 basic foods common to SAYCARE participating centers and includes some typical foods, totaling in 67 food groups for São Paulo, for example. Similar to the food booklet, the fruits and vegetables were questioned separately in four different groups such as fresh fruit, fresh fruit juice, raw vegetables, boiled vegetables, and vegetable soup. These groups were defined in nine frequencies of intake, as following: (1) Never/less than once a month; (2) 1–3 times per month; (3) once a week; (4) 2–4 times per week; (5) 5–6 times per week; (6) once a day; (7) 2–3 times per day; (8) 4–5 times per day; and (9) 6 or more times per day [20]. In addition, it was questioned whether the research participant consumed any type of supplement with vitamin A, C, and/or E.

The number of portions of fruits and vegetables consumed was calculated in grams per day, by the sum of the frequency of the fruits and vegetables specified in the FFQ, transformed into frequency of daily consumption, and multiplied by the size of the portion consumed. 

For example, if the parent/guardian answered that his/her child usually eats one portion of fruit per day and one portion of vegetables 2–3 times per day, the total fruit and vegetable daily intake was calculated as [fruit: 80 (medium portion) × 1 (once a day) = 80 g/day] + [vegetable: 50 (medium portion) × 2.5 (2–3 times per day) = 125 g/day]. Therefore, the total fruit and vegetable daily intake were (80 + 125) 205 g per day.

#### 3.5.2. 24-h Dietary Recall (24 h-DR)

The 24 h-DR is an open questionnaire on all food consumed in the previous day, at all times and meals, and it was answered three times with 15 days-interval among them (2 non-consecutive weekdays—by trained nutritionists, 1 weekend day—by parents/guardians, with the help of the child, and with the instructions provided).

The intake of foods and beverages reported in the 24 h-DR in home measures were calculated in standard measures (grams or milliliters), with the assistance of the Table for Evaluation of Food Consumption in Domestic Measures [22]. Two research nutritionists (TSC and TRU) with fluent comprehension, speaking and writing in Portuguese and Spanish languages translated the food items from the 24 h-DR in Portuguese to Spanish because the food software used in this study is in the Spanish language. This software was developed to evaluate energy and nutrient intake from Ibero-American food consumption surveys. The food composition databases included in it are as follows: Spanish Food Composition Database (Bedca); National Nutrient Database for Standard Reference Release (USDA); Ibero-American Foods Database. Moreover, some typical Brazilian foods were inserted into this software, considering the Brazilian Food Composition Table (TACO) [23]. Therefore, data from 24 h-DR were entered in the software, and tabulated in an Excel spreadsheet, for further analyzes in multiple source method (MSM). The MSM combines information of frequency of intake (from the FFQ) and the mean intake (from the 3 days of the 24 h-DR) to better predict usual dietary intake [24]. The MSM program is a web-based tool with open access at the website https://nugo.dife.de/msm/. At the MSM website, we uploaded an Excel spreadsheet with a structure suitable for MSM analysis, including the habitual frequency of fruit and vegetable daily intake (from the FFQ), and the fruit and vegetable intake (in grams per day) from each of the three days of the 24 h-DR. We defined the MSM Model structure by specifying the response variable as the 24 h-DR, and we defined the MSM regression model using the variable “sex”.

We considered all fruits, fresh fruit juices, vegetables, vegetables soup and tomato sauce in the fruit and vegetable intake, in both dietary assessment methods.

### 3.6. Blood Processing and Analyzes

Families and children were instructed to keep the research participant fasted (10 h) for blood collection. A snack was offered to the child after the blood examination. Experienced nurses collected 10 mL of blood in the antecubital vein, which were distributed in three tubes (EDTA, heparin, serum).

The blood samples of complete blood count and total cholesterol were sent to the central laboratory of the Clinical Hospital from Medical School/University of São Paulo (USP) for processing and analyze, in the following order: (1) Collection of tube with coagulation accelerator; (2) rest (10 min) at room temperature; (3) centrifugation (15 min at 3000 rpm); (4) serum storage at −20 °C [17]. The requirements for pre-analytical and analytical stages are standardized following the guidelines of the World Health Organization [25] and the Clinical Laboratory Standards Institute [26], to diminish errors and variability. Total cholesterol was classified following the Brazilian consensus for the normalization of laboratory determination, 2016 [27], and the c-reactive protein was classified according to Brazil, 2007 [28].

For plasma vitamin analysis, we used the following biomarkers: Retinol, β-carotene, ascorbic acid, and α-tocopherol. Such vitamins are highly sensitive to light, and ascorbic acid is a particularly labile vitamin [29]. Thus, these analyses require a specific methodology and adapted to the conditions, mainly, of light and temperature. Therefore, we performed a careful and sophisticated blood examination only in São Paulo, because it was the only center from the SAYCARE study with a laboratory expert in this type of analysis—the Micronutrient Laboratory of the School of Public Health of the University of São Paulo (FSP/USP). It is important to emphasize that the blood examination was done at schools, with the tubes coated with aluminum paper to protect from light, and the blood samples were immediately transported by a company specialized in the transport of biological samples (in adequate conditions of light and temperature), in a maximum time period of 40 min from the schools to the laboratory for analysis, in order to minimize the oxidation of vitamins. These samples were stored in its tube, protected from light, at −20 °C (for one month), and later at −80 °C. All analyses were performed in duplicate, using high-performance liquid chromatography (HPLC). The liquid chromatography equipment used was an LC-20AT HPLC system (Shimadzu, Inc., Chiyoda-Ku, Tokyo, Japan), equipped with an SIL-20AC automatic injector, CBM-20A controller, CTO-20A column oven (40 °C), and SPD-M20A diode array detector, using a Luna C18 separation column (250.0 × 4.6 mm, 5 µm particle size; Phenomenex, Torrance, CA, USA).

Plasma concentrations of retinol, β-carotene, and α-tocopherol were assayed using the methodology proposed by Arnaud et al. (adapted) [30], with 400 μL of plasma collected and stored in the absence of light (coated with aluminum paper). Retinol concentrations were detected at a retention time of 2.5 min; β-carotene in 6.7 min; and α-tocopherol in 4.5 min. Thus, the total time of the chromatographic run was 8 min (approximately). The ascorbic acid was analyzed according to the methodology of Robitaille and Hoffer (adapted) [31], with 200 μL of plasma collected and stored in the absence of light (coated with aluminum paper), and analyzed on the same day of thawing. The following cut-off points were considered acceptable for plasma vitamin level if: Retinol ≥ 0.70 μmol/L [32]; α-tocopherol ≥ 16.2 μmol/L [33]; ascorbic acid ≥ 26.1 μmol/L [31].

### 3.7. Statistics

We used the Stata 14 (Stata Corp., College Station, TX, USA) program for statistical analyses. We performed sensitivity analysis to detect possible selection biases caused by missing values from the FFQ and maternal education level, and no difference was found. After the calculation of fruit and vegetable intake in grams per day for both dietary assessment methods and the calculation of total energy intake in kcal per day for the FFQ, we excluded children who showed a physiologically implausible energy intake (less than 500 or over 4000 kcal/day) in the FFQ [34]. The Shapiro–Wilk test was used to investigate the distribution of variables and the Wilcoxon to test differences between reported intakes between the FFQ and the mean usual intake estimated by the MSM. Our outcomes were the correlation coefficients between the dietary assessment methods and plasma vitamins, which we calculated using multiple linear regression with their corresponding 95% confidence intervals (considering *p* < 0.05 as significance level). The regressions were adjusted based on the literature and tested in the study data. Consequently, the variables that remained in the regression model were sex, total energy intake, total cholesterol (α-tocopherol), and maternal education for the FFQ; and sex, waist circumference, total cholesterol (α-tocopherol), and maternal education for the MSM.

In addition, we calculated the sample-weighted pooled correlation coefficient using the meta-regression analysis, intending to analyze the combination of all plasma vitamins together and its correlation with the group of fruit and vegetable intake, estimated by each dietary assessment methods. As children were from the same sample (no heterogeneity was found among them), we used the fixed model in the meta-regression analysis. We estimated the strength of the agreement of the correlation coefficients cutoff points using the classification for dietary assessment methods: Weak < 0.20, fair = 0.21 to 0.40, moderate = 0.41 to 0.60, good = 0.61 to 0.80, and >0.80, very good [35].

Furthermore, we applied the receiver operating characteristics (ROC) curve analysis to calculate the accuracy of the dietary assessment methods with plasma vitamins (as a binary outcome according to the reference for the age group). ROC curve provides the whole spectrum of accuracy, specificity and sensitivity values for all possible cut-off points. We verified the accuracy of both dietary assessment methods using the ROC curve for fruit and vegetable intake as a continuous variable. Moreover, as an explanatory analyses, we investigated the percentage of accuracy within the recommendation established by the American Heart Association for children and adolescents aged 2–19 years [1], regarding the intake of 4 to 5 cups of fruits and vegetables per day. Since this recommendation is wide and there is no agreed definition on what constitutes the number of grams these cups represent, we considered that each cup is equivalent (on average) to 80 g. Thus, multiplying the cups into grams, we categorized the American Heart Association recommendation of fruits and vegetables for children in minimum (320 g/day), mean (360 g/day), and maximum (400 g/day). Then, ROC curves were run against plasma vitamins separately, and with these vitamins together (combined in a score of plasma vitamins recommendations), to test whether this combination could improve accuracy, since we are validating the fruit and vegetable intake together, and not individually by type of vitamin. As there is no reference for β-carotene levels in plasma, only ascorbic acid, α-tocopherol and retinol were summed up in this score. Children who accomplished one recommendation in plasma levels of the vitamin received one point, and the ones who did not, received zero points. Then, the children who accomplished two or more recommendations in plasma levels of the vitamins were computed as “good plasma levels of the vitamins score”, and the ones who accomplished only one recommendation or none of the recommendations were computed as “insufficient plasma levels of the vitamins score”. The area under the curve (AUC) was determined from plotting sensitivity versus one—the specificity of a test as the threshold varies over its entire range [36], and it to determine the overall accuracy of each dietary assessment methods (AUC ≥ 0.5 was considered to have diagnostic value, the larger the area, the larger the value) [37].

### 3.8. Funding

The SAYCARE study was mainly supported by the Brazilian Government through the National Council of Technological and Scientific Development (CNPq; proc. 471266/2013-2) and by the São Paulo Research Foundation (FAPESP; proc. 2014/11468-6). This validity study of children’s fruit and vegetable intake using plasma vitamins A, C, and E was financed by FAPESP through Ph.D. scholarship number: 2016/13922-1.

### 3.9. Ethics

This study was approved by the Research Ethics Committee of Medical School of the University of São Paulo, under research protocol no. 2,022,542. The parental consent form was signed by all the parents/guardians of the research participants, and all children gave verbal agreement to participate in the study.

## 4. Results

### 4.1. Participant Characteristics

Of the 60 children (6 to 10 years) who were identified at schools, 57 of them were interested in participating in the study and signed the parental consent form, 55 completed the blood examination, but four of these children did not have enough plasma samples for vitamin analysis. Thus, 51 children completed all plasma vitamins analyses. Of these children, we excluded two children with physiologically implausible energy intake according to the FFQ, and four children were excluded because they showed blood concentrations of c-reactive protein over 10.0 mg/dL(these levels are suggestive of active inflammation/infection that could affect the bioavailability of the vitamins in plasma) [28].

**Figure 1:**
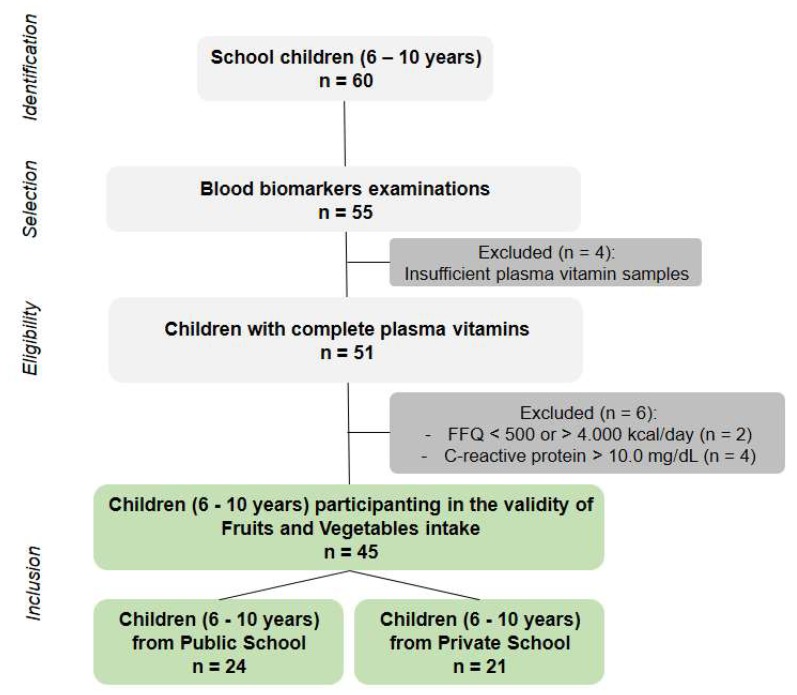
Flow chart of children (6–10 years) participating in the validity of questionnaires ranking fruit and vegetable intake with plasma vitamins.

The selection of participating children (n = 45) is presented in the flow chart (Figure 1), and their general characteristics in Table 1. Females represent 53.3% of the sample, the mean age was 8.3 years, 33.3% were with overweight or obesity, and 24.4% were classified with high total cholesterol levels. We questioned about supplementation, but no child was taking supplements with vitamin A, C, and/or E.

### 4.2. Fruit and Vegetable Intake

**Figure 2:**
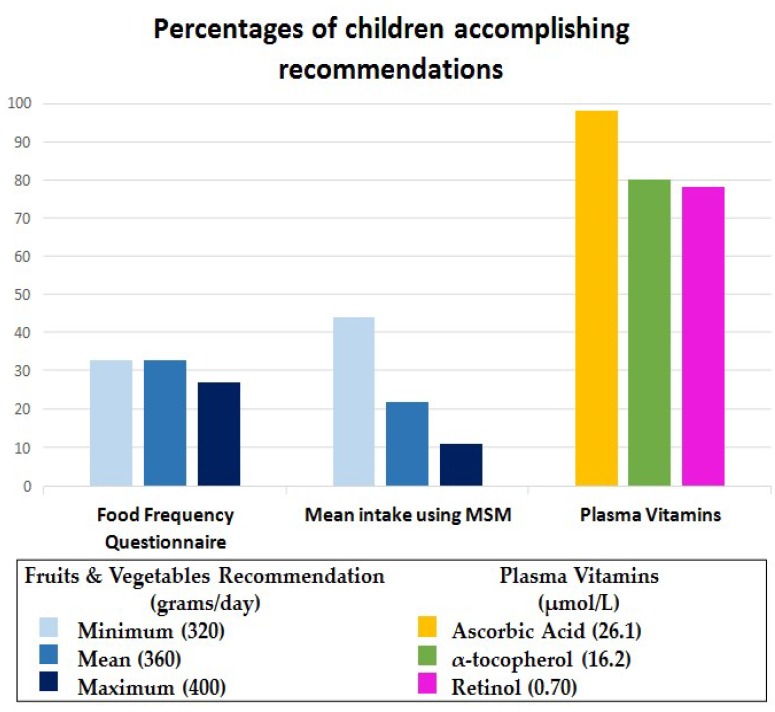
FFQ: Food frequency questionnaire; MSM: Multiple source method (the combination of frequency of intake with 3 days of 24-h Dietary Recall). These prevalences are based on adherence to the following recommendations: Fruits and vegetables [1]; ascorbic acid [31]; α-tocopherol [33]; retinol [32]. Percentages of accomplished recommendations for children’s (6–10 years) fruit and vegetable intake and plasma vitamins.

The mean usual fruit and vegetable intake estimated by the SAYCARE FFQ (261.2 g/day) was similar to the mean intake (301.7 g/day) calculated by the MSM (using the combination of three days of 24 h-DR with the frequency of intake). The difference is that the MSM showed more precise 95% confidence intervals, and the distribution of the fruit and vegetable intake is parametric (Supplementary Figure S1). No significant difference was found neither between the type of schools nor between fruits and vegetables separately. Therefore, the analyses were conducted with both schools together and with fruits and vegetables together, as well (Table 2). Regarding the 24 h-DR, the children/parent/guardian reported 52 different types of fruits and vegetables (Supplementary List S2). The fruit and vegetable intake is presented as a continuous variable in grams per day, and as explanatory analyses, we investigated the percentage of children who accomplished the fruit and vegetable recommendation by the American Heart Association [1] calculated using the MSM, and the recommendations for plasma ascorbic acid [31], α-tocopherol [33] and retinol [32] (Figure 2).

### 4.3. Correlation Coefficients

**Figure 3:**
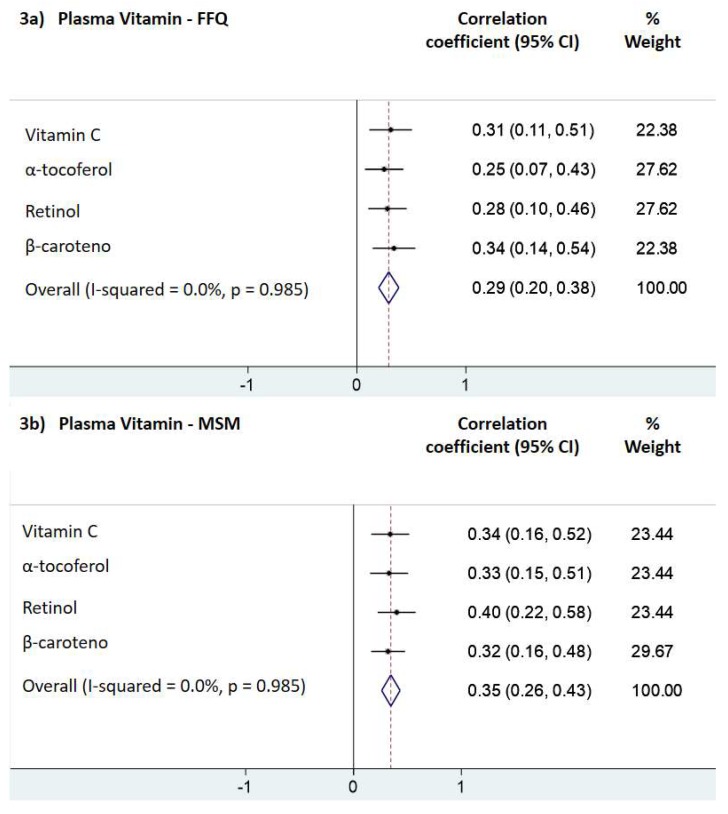
95% CI: 95% Confidence interval; FFQ: Food frequency questionnaire; MSM: Multiple source method (the combination of frequency of intake with 3 days of 24-h Dietary Recall). (**a**,**b**) Correlation coefficients from multiple linear regression for children’s (6–10 years) fruit and vegetable intake estimated by the FFQ versus plasma vitamins (**a**); the MSM versus plasma vitamins (**b**).

Figure 3 presents the correlation coefficients from multiple linear regression for fruit and vegetable intake and each biomarker separately, and all biomarkers together calculated by the meta-regression analysis [Figure 3a for the FFQ; Figure 3b for the MSM (the combination of frequency of intake with 3 days of 24-h dietary recall)].

### 4.4. Accuracy of Dietary Assessment Methods

The percentage of accuracy from the ROC curves of fruit and vegetable intake estimated by FFQ and by MSM, according to the type of vitamin is shown in Table 3. The fruit and vegetable intake is presented as a continuous variable, and as an explanatory analyses, we investigated the percentage of accuracy within the minimum (320 g), medium (360 g), and maximum (400 g) fruit and vegetable recommendation by the American Heart Association for children and adolescents (2–19 years) [1]. It indicates that when vitamins are combined in a score, the recommendation of 320 and 360 g of fruits and vegetables showed the highest accuracy (67.9%), for the FFQ; and the recommendation of 360 of fruits and vegetables showed the highest accuracy (61.9%), for the MSM.

## 5. Discussion

This study aimed to validate the FFQ and the combination of the frequency of intake from the FFQ with the 24 h-DR (mean of 3 days), both designed for the SAYCARE Study, for children’s fruit and vegetable intake. The reproducibility of the SAYCARE FFQ for this food group was previously tested by comparing it against the 24 h-DR [21], with fair agreement in children. Consequently, now we are conducting the validity the SAYCARE FFQ with a superior and objective method, the plasma vitamins. We found pooled correlation coefficients of 0.29 (95% Confidence Interval: 0.20–0.38) and 0.35 (95% Confidence Interval: 0.26–0.43) for the reported fruit and vegetable daily intake estimated by the FFQ and the MSM, respectively. These findings are comparable to other validity studies assessing fruit and vegetable consumption in children [3,38], and also in adolescents [39]. The MSM showed a slight increase in the correlation coefficients for each plasma biomarker separately, and in the pooled correlation coefficient when compared to the correlations estimated by the FFQ. However, all correlation coefficients from multiple linear regression (separately or together) were in the acceptable range according to the literature [35]. Correlations among plasma vitamins and estimates of dietary intake measured by questionnaires are expected to be considerably lower than 1.0, even when fruit and vegetable intake is perfectly assessed because plasma vitamins are also influenced by numerous variations in absorption and metabolism [13]. Thus, it suggests that both FFQ designed for the SAYCARE Study or the combination of 3 days of 24 h-DR with the frequency of intake using the MSM, are valid dietary methods for ranking children’s fruit and vegetable intake.

These results are important to support the possibility of using only the FFQ designed for the SAYCARE Study to rank children’s fruit and vegetable intake. Consequently, financial and timing resources can be optimized, since there is no need to apply the 24 h-DR (three times), the presence of a trained nutritionist in the fieldwork to apply the 24 h-DR, or to dedicate labor-intensive time and efforts for analyzing these data from the 24 h-DR and calculating them with the MSM [40].

### 5.1. Schoolchildren

We were able to reach more children participating in this validity study (n = 45) than our sample size calculation (n = 30). The distribution of sex and school was similar within the sample. Furthermore, the population from São Paulo is very diverse in cultural and ethnic aspects, and it might also reflect in the variety of fruits and vegetables consumed [41]. On the other hand, 33.3% of children were with overweight or obesity [42], and 24.4% were classified with high total cholesterol levels [27]. These two facts were considered in the analyses (adjustments for waist circumference and total cholesterol).

Children from public school represent 46.7% of the sample; yet, the majority of mothers had a university degree. Even though maternal education was treated as a confounder in the analysis, it is a limitation of our study, as it might have improved the results found. Besides, 13 mothers did not answer this variable, which might bias the results as well [43]. But after sensitivity analysis, no difference was found between them.

### 5.2. Subjective Dietary Assessment Methods

FFQs are indicated as the most feasible approach for examining long term intakes on hard endpoints, when they are complemented by nutritional related biomarkers, such as plasma vitamins [44]. The SAYCARE FFQ was designed to be culturally adapted to the South American centers participating in the Study and specific to the pediatric population, to be informative regarding some food groups and dietary patterns, but one of its limitations is the food combination (e.g., different types of fruits and vegetables all together) [44]. However, this may not make a significant difference on validity studies ranking fruit and vegetable intake, since the recommendation of this food group is general, and does not require specification of the fruit/vegetable type [1].

While potentially useful and inexpensive, the FFQ is also prone to other limitations and errors. The FFQ developed for the SAYCARE Study is restricted to the consumption of the previous three months, and omissions can occur possibly due to the fixed food list [20]. Moreover, to answer the FFQ it is necessary that the reporter is literate, and to have cognitive and memory skills to estimate the habitual food consumption [45]. Despite these limitations, a food booklet was developed to assist in the estimation of fruits and vegetables portions. Furthermore, a trained nutritionist applied two of the three 24 h-DR, and we used the MSM to combine the 24 h-DR with the frequency of intake from the FFQ, trying to improve the estimates of fruit and vegetable intake [46].

Another limitation is that we considered fresh fruit juices, vegetables soup and tomato sauce in both dietary assessment methods. This might have improved the fruits and vegetables grams, because of the water content in these preparations. However, as we are validating the consumption in school-age children (6–10 years), these preparations are commonly consumed by this age range, especially in São Paulo, where children are culturally accustomed to drinking fresh fruit juice at lunch, dinner, and sometimes also together with a snack.

### 5.3. Combination of Biomarkers

As the American Heart Association recommends the intake of 4 to 5 cups of fruits and vegetables per day for children, we aimed to validate the dietary assessment methods considering all fruits and vegetables together (as this recommendation informs). Therefore, we decided to analyze the combination of the plasma biomarkers together as well, for two main reasons. Primarily because the combination of fruits and vegetables is a complex food group, and the bioavailability of phytochemicals vary noticeably between types of fruits or vegetables [47], even in the same fruit or vegetable it may vary according to conditions of storage, transport, cooking method and also to content of the nutrient in the geographic region where it was planted [13]. Secondly, many factors might influence the biomarker response to fruit and vegetable intake, including inter-individual variation in digestion, absorption, and metabolism [13]. Some of this variation might be related to genetic factors, but environmental factors such as body composition also influence the concentration of the biomarkers [48]. That is the main reason for adjusting the analyses for waist circumference since it correlates better with central adiposity in the body. Thus, other studies also suggested that the combination of biomarkers enhanced the prediction of fruit and vegetable intake [29,47], because, at this moment of validity study, we don’t require a complete understanding of the plasma vitamins individually, but whether their overall combination (including the interrelationships cited above) gives a complementary information to validate the questionnaires ranking children’s fruit and vegetable intake [44].

### 5.4. Accuracy of Dietary Assessment Methods

Surprisingly, almost 80% of the sample accomplished the recommendations for plasma vitamins. However, mean fruit and vegetable intake were below the recommendation (320 to 400 g per day) [1] in both dietary assessment methods. The accurate assessment of children’s fruit and vegetable intake is challenging, and questions remain regarding the best quantity of fruits and vegetables that children should eat daily [29]. For example, Figure 2 shows that when we considered the minimum recommendation (320 g/day), only 33% and 44% of participants reached this reference when estimated by the FFQ and the MSM, respectively. These percentages decreased for the maximum recommendation (400 g/day), in both dietary assessment methods. It indicates that both methods might be used to rank the children’s fruit and vegetable intake, but not to translate it into absolute quantities of intake. Another possible explanation for the difference between lower percentages of fruit and vegetable intake and higher percentages of plasma vitamin levels is that many food products for children are fortified with vitamins [38], and even though children are not eating the recommended amount of fruits and vegetables, their plasma vitamins levels are mostly adequate due to vitamin fortification of commercial food products [13,40]. A study carried out in Finland to assess the relationship between children’s fruit and vegetable intake and blood biomarkers also found that the high consumption of fortified foods reflected higher serum carotenoids levels [38]. Furthermore, a good vitamin profile in plasma might also reflect a better dietary variety and quality [49].

Moreover, there is no universally agreed upon recommendation for fruit and vegetable intake specifically for children from 6 to 10 years of age. Even though the American Heart Association targets children and adolescents (2–19 years), its fruit and vegetable recommendation (4–5 cups a day) [1] is different than the recommendation from the USDA for children between 4–8 and 9–13 years of age (2.5 to 5 cups a day) [50]. In addition, the absence of an average portion size for fruit and vegetable that is appropriate for children is an important limitation to translate these recommendations into grams per day.

### 5.5. Adjustment for Confounders

As previously mentioned, numerous factors may influence the assessment of fruit and vegetable intake with subjective methods (e.g., socially desirable answers and errors in food composition databases) [40] and also the concentration of vitamins in plasma (e.g., genetic, environmental, lifestyle, homeostatic) [29]. Thus, it is extremely important that studies of validity of children’s fruit and vegetable intake using plasma vitamins adjust the analyzes for confounders [51]. Dietary assessment questionnaires for children younger than 10 years of age are usually answered by parents [4], therefore maternal education is a paramount adjustment, especially in low and middle-income countries [43]. Waist circumference is also useful, once it is an indicator of central adiposity that may influence the bioavailability of vitamins in plasma [13]. Moreover, α-tocopherol (precursor of vitamin E) should be adjusted for blood cholesterol, because vitamin E is transported in blood lipoproteins and is strongly related to blood cholesterol [13]. Finally, even though in our sample we did not find differences between sex, it is crucial that studies adjust for sex due to different behaviors between girls and boys [3].

### 5.6. Strengths and Limitations

As far as we know, this is the first study validating usual fruit and vegetable intake in children from middle-income countries, such as Brazil. One limitation of our study is that we were not able to include the β-carotene in our accuracy analyzes, because there is no reference value for βa-carotene in blood. The β-carotene and retinol are precursors of vitamin A, and they are important for health, growth, and development, as vitamin A deficiency can be associated with increased risks of xerophthalmia, low bone mineral density, immune dysfunction, infectious morbidities and also mortality in children [52]. Therefore, it is crucial that researchers conduct an explicit, objective, and transparent study to define reliable β-carotene reference values [52]. Also, we suggest that more studies need to verify whether the reference for fruit and vegetable intake should be the same for all populations age, since children have significantly different necessities and behaviors than adults or elders, for example.

## 6. Conclusions

Our findings suggest that the FFQ designed for the SAYCARE Study is a useful tool for ranking children’s fruit and vegetable intake with acceptable accuracy, and with relative precision level as the MSM (the combination of frequency of intake with the 3 days of 24 h-DR) when compared to plasma vitamins. Furthermore, both methods showed to be valid tools for ranking fruit and vegetable intake in children between 6 to 10 years from the SAYCARE Study.

## Figures and Tables

**Table 1:** Characteristics of the subsample of children (6–10 years) from the SAYCARE (South American Youth/Child Cardiovascular and Environmental) study with plasma vitamins. São Paulo, Brazil. 2017.

	n	% or Mean	(95% CI)
Sex (*n* = 45)			
**Female**	24	53.3	(38.3–67.8)
**Male**	21	46.7	(32.2–61.7)
Age in years (n = 45)		8.3	(7.9–8.6)
Body Mass Index (kg/m^2^) * (*n* = 45)			
**Underweight**	1	2.2	(0.3–15.1)
**Healthy weight**	29	64.4	(49.0–77.4)
**Overweight**	10	22.2	(12.1–37.2)
**Obesity**	5	11.1	(4.5–24.7)
Waist Circumference (cm) (*n* = 45)		62.1	(59.0–65.3)
Total Cholesterol (*n* = 45) **			
**Healthy** (<170 mg/dL)	34	75.6	(60.4–86.2)
**High** (>170 mg/dL)	11	24.4	(13.8–39.6)
C-reactive protein (mg/dL) (*n* = 45)		0.8	(0.4–1.2)
School (*n* = 45)			
**Public**	24	46.7	(32.2–61.7)
**Private**	21	53.3	(38.3–7.8)
Maternal Education Level (*n* = 32) !			
**Incomplete high school**	3	9.4	(2.9–26.7)
**High school**	12	37.5	(22.0–56.1)
**Technical**	1	3.1	(0.4–20.9)
**University degree**	16	50.0	(32.5–67.5)

95% CI: 95% Confidence Interval. kg/m^2^: Kilograms per square meter. mg/dL: Milligrams per deciliters. * Classified according to Cole et al., 2007 [19]. ** Classified by the Brazilian consensus for the normalization of laboratory determination, 2016 [27]. ! Missing data.

**Table 2:** Fruit and vegetable intake and plasma vitamins concentration in a subsample of children (6–10 years) from the SAYCARE Study. São Paulo, Brazil. 2017.

	Total		Public School			Private School
	n	Mean (95% CI)	n	Mean (95% CI)	n	Mean (95% CI)
**Fruit and Vegetable Intake (grams/day)**						
FFQ	45	261.2 (195.1–327.3)	21	225.9 (139.4–312.4)	24	292.1 (189.5–394.7)
Mean intake using MSM	45	301.7 (277.2–326.2)	21	309.0 (278.1–340.0)	24	295.3 (256.2–334.4)
**Plasma Vitamins (μmol/L)**						
Ascorbic Acid	45	80.9 (66.5–95.4)	21	78.8 (64.9–92.8)	24	82.8 (57.5–108.1)
α-tocopherol	45	21.8 (20.2–23.3)	21	21.1 (18.3–23.9)	24	22.3 (20.5–24.1)
Retinol	45	0.94 (0.84–1.03)	21	0.88 (0.76–1.0)	24	0.98 (0.84–1.12)
β-carotene	45	0.32 (0.26–0.39)	21	0.31 (0.24–0.39)	24	0.33 (0.23–0.44)

95% CI: 95% Confidence interval; FFQ: Food frequency questionnaire; MSM: Multiple source method (the combination of frequency of intake with 3 days of 24-h Dietary Recall).

**Table 3:** Percentage of accuracy from receiver operating characteristics (ROC) curve of fruits and vegetables daily intake estimated by FFQ and by MSM according to the type of plasma vitamin. São Paulo, Brazil. 2017.

Fruits and Vegetables Daily Intake	Plasma Vitamins			
	Ascorbic Acid	α-Tocopherol	Retinol	Sum of Vitamins (≥2 Points)
**FFQ, accuracy (%)**				
Continuous	47.7	51.5	64.2	-
Minimum (320 g/day)	67.1	50.0	62.7	67.9
Mean (360 g/day)	67.1	50.0	62.7	67.9
Maximum (400 g/day)	63.6	52.8	58.6	64.3
**Mean intake using MSM, accuracy (%)**				
Continuous	65.9	71.3	30.1	-
Minimum (320 g/day)	72.7	56.9	31.4	56.0
Mean (360 g/day)	61.4	63.9	33.1	61.9
Maximum (400 g/day)	55.7	56.9	34.0	56.0

FFQ: Food frequency questionnaire; MSM: Multiple source method (the combination of frequency of intake with 3 days of 24-h Dietary Recall).

## Supplementary Materials

The following are available online at https://www.mdpi.com/2072-6643/11/8/1815/s1, Figure S1: Fruits and vegetables intake distribution calculated by the multiple source method (the combination of 3 days of 24-h Dietary Recall with the frequency of intake) in children. List S2: Different types of fruits and vegetables reported at the 24-h Dietary Recall.
