# Supplementary material for: The Validity of Children’s Fruit and Vegetable Intake Using Plasma Vitamins A, C, and E: The SAYCARE Study
Source: Nutrients. 2019 Aug 6;11(8):1815. doi: 10.3390/nu11081815 (PMC6722842; doi:10.3390/nu11081815)
Supplement: Supplementary file 1 [file nutrients-11-01815-s001.zip › supplementary files_zip/Supplementary List S2_FV.pdf]

Supplementary List S2 - Fruits and vegetables reported at the 24-hour dietary recall

List S2. Different types of fruits and vegetables reported at the 24-hour dietary recall, in Portuguese and the respective translation in English. São Paulo, Brazil. 2017

**Fruits**	
**Portuguese**	**English**
1. Abacate	1. Avocado
2. Água de coco natural	2. Natural coconut water
3. Ameixa	3. Plum
4. Banana	4. Banana
5. Laranja	5. Orange
6. Maçã	6. Apple
7. Mamão formosa	7. Papaya "*Formosa*"
8. Mamão Papaia	8. Papaya
9. Manga	9. Mango
10. Melancia	10. Watermelon
11. Melão	11. Melon
12. Mexerica	12. Tangerine
13. Morango	13. Strawberry
14. Pêra	14. Pear
15. Pêssego	15. Peach
16. Salada de frutas	16. Fruit Salad
17. Suco de cenoura natural	17. Natural Carrot Juice
18. Suco de laranja natural	18. Natural Orange Juice
19. Suco de limão natural	19. Natural lemon juice
20. Tomate	20. Tomato
21. Uva roxa	21. Purple grape
22. Uva verde	22. Green grape

List S2. Continue

Vegetables	
**Portuguese**	**English**
1. Abóbora	1. Pumpkin
2. Abobrinha	2. Zucchini
3. Acelga	3. Chard
4. Alface	4. Lettuce
5. Alho	5. Garlic
6. Aspargos	6. Asparagus
7. Batata assada	7. Roasted potatoes
8. Batata cozida	8. Baked potato
9. Batata doce	9. Sweet potato
10. Berinjela	10. Eggplant
11. Beterraba	11. Beetroot
12. Bróbolis	12. Broccoli
13. Canja de galinha	13. Vegetable and chicken soup
14. Cebola	14. Onion
15. Cenoura	15. Carrot
16. Chuchu	16. Chayoti
17. Cogumelo	17. Mushroom
18. Couve	18. Cabbage (green col)
19. Ervilha	19. Peas
20. Espiga de milho	20. Corn on the cob
21. Espinafre	21. Spinach
22. Feijão Branco	22. White beans
23. Feijão carioca	23. Brown beans
24. Feijão preto	24. Black beans
25. Gengibre	25. Ginger
26. Lentilha	26. Lentil
27. Milho	27. Maize, corn
28. Pepino	28. Cucumber
29. Pimentão	29. Pepper
30. Repolho	30. Cabbage
