# Supplementary figures and images for: The Validity of Children’s Fruit and Vegetable Intake Using Plasma Vitamins A, C, and E: The SAYCARE Study
Source: Nutrients. 2019 Aug 6;11(8):1815. doi: 10.3390/nu11081815 (PMC6722842; doi:10.3390/nu11081815)

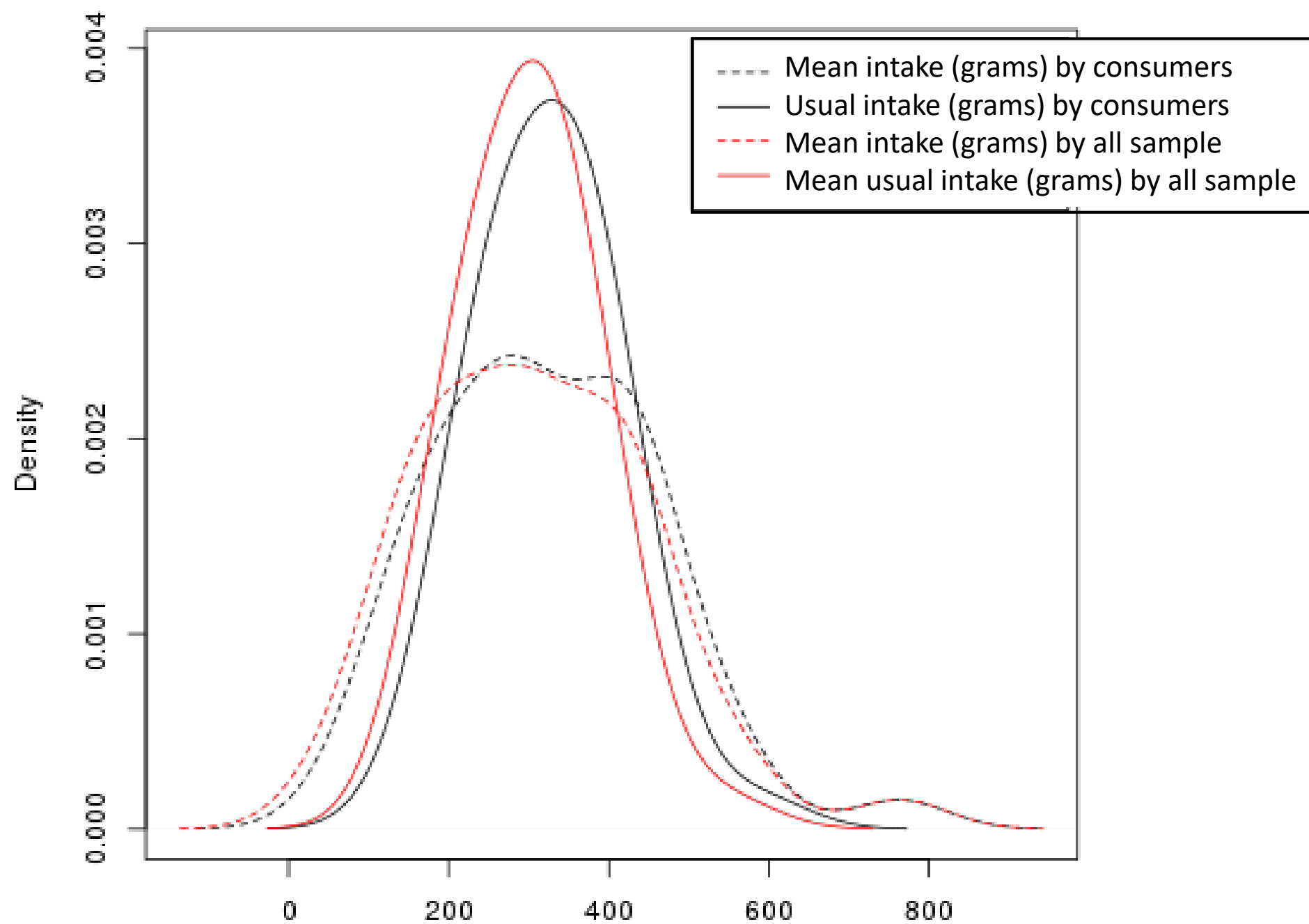

N = 45 Bandwidth = 59.63

Supplement: Supplementary file 1 [file nutrients-11-01815-s001.zip › supplementary files_zip/Suplementary Figure S1.pdf]
